# Circumcised men’s perceptions, understanding and experiences of voluntary medical male circumcision in KwaZulu-Natal, South Africa

**DOI:** 10.4102/safp.v62i1.5083

**Published:** 2020-05-19

**Authors:** Celenkosini T. Nxumalo, Gugu G. Mchunu

**Affiliations:** 1 Department of Nursing, School of Nursing and Public Health, University of KwaZulu-Natal, Durban, South Africa

**Keywords:** voluntary medical male circumcision, barriers, motivators, circumcised male candidates, experiences, perceptions, HIV prevention, uptake

## Abstract

**Background:**

KwaZulu-Natal, South Africa, has rolled out voluntary medical male circumcision (VMMC) in response to recommendations that regions with a high human immunodeficiency virus (HIV) prevalence adopt VMMC as an additional HIV prevention strategy. There is a paucity of South African data on the motivators, barriers and experiences of adult male candidates regarding VMMC. This study was conducted to analyse circumcised men’s perceptions, understanding and experiences of VMMC in KwaZulu-Natal, South Africa.

**Methods:**

A qualitative phenomenographic design was used. Ethical clearance was obtained from the Biomedical Research Ethics Committee of the University of KwaZulu-Natal (BE 627/18). Data were collected from 12 circumcised male candidates. Individual interviews were conducted and recorded by using an audiotape. Data were transcribed verbatim and analysed manually.

**Results:**

Participants’ perceptions regarding VMMC are health related and appear to be the motivators for the uptake of medical circumcision. Circumcised men in this study appeared to misunderstand VMMC in terms of healing and performance time and the nature of the procedure. Negative experiences in terms of quality of care received were reported.

**Conclusion:**

The study findings imply that practice interventions to promote demand generation for VMMC in KwaZulu-Natal, South Africa, should incorporate the perceptions and experiences of male candidates regarding the procedure. Tailored messaging to address misunderstanding related to the nature of VMMC should also be provided. Regular in-service training on standardised VMMC implementation practices should be provided to ensure the delivery of optimum quality VMMC services.

## Introduction

Voluntary medical male circumcision (VMMC) is one of the core evidence-based interventions prioritised by the World Health Organization (WHO) and the Joint United Nations Programme on HIV and AIDS (UNAIDS) for countries where there is a high prevalence of HIV.^1,2,3^ Three randomised controlled trials have proved that VMMC reduces the risk of transmission of HIV by up to 60%, thus offering partial protection against the spread of HIV infection.^4,5,6^

Globally, sub-Saharan Africa is the leading region in terms of the incidence and prevalence of HIV infection-related mortality and morbidity.^7^ South Africa, particularly the KwaZulu-Natal (KZN) province, contributes the most in terms of the burden of HIV infection. By the end of 2018, approximately 7.7 million people had been living with HIV in South Africa, there were 240 000 new infections and 71 000 people died from HIV or AIDS related illnesses.

In South Africa, VMMC was rolled out throughout the country in 2010 and by the end of 2014, 1.9 million male candidates had received VMMC.^8^ Although statistical data on the uptake of medical circumcision show significant strides in the number of medical circumcisions performed to date, there appears to be a low demand for VMMC among older male candidates, aged 15–49 years,^9^ particularly in the KZN province. Reaching 80% coverage in this age group is crucial as they have higher HIV infection rates.^10^

Modelling studies on VMMC have reported that facilitating the uptake of VMMC to reach the coverage of 80% of male candidates aged 15–49 years could prevent approximately 80 000 new infections per year and potentially contribute to an HIV-free generation. This in turn could result in an estimated cost saving of 16 billion United States dollars in HIV infection-related healthcare expense.^11,12^

Studies on the acceptability of medical circumcision among men have revealed that VMMC programmes need to consider contextual factors, including people’s perceptions and understanding of male circumcision, to increase the demand for the procedure.^13,14,15,16^ Recent literature on the scale-up of VMMC has shown that several factors hinder and influence the uptake of VMMC by male candidates; these include psychosocial factors, health system factors, the influence of female partners and religious and traditional factors.^9,17,18^ In the KZN province, the issue of circumcision being culturally discouraged in Zulu communities is one of the major barriers to the uptake of VMMC.^19^

Although many studies have been conducted on the uptake of VMMC in sub-Saharan Africa,^19,20,21,22,23^ there is a dearth of such research among older circumcised men, especially in KZN, South Africa, which is also the province with least circumcisions.

This article presents findings on circumcised men’s perceptions, understanding and experiences of VMMC, which was one component of a study to identify factors that hinder or promote the uptake of medical circumcision by men in KZN, South Africa. The article focuses solely on circumcised men as the other parts of the larger project have focused on barriers to VMMC in uncircumcised men.

## Methods

### Design

The study used a qualitative phenomenographic design with a view to developing an explanatory model to inform inclusion of contextual factors in the VMMC programme in KZN, South Africa. Phenomenography, as a design, emerges from an empirical study by Marton and Booth to discover the qualitatively different ways in which people experience, conceptualise and understand a particular phenomenon.^24^ The main purpose of phenomenography is to provide and describe a variety of perceptions, experiences and understanding about a particular phenomenon. The basic assumption of phenomenography is that an individual does not experience a phenomenon in the same way as another; therefore, a single person may not necessarily be able to explain all aspects of a concept.^25^

### Study setting

The study was conducted in six primary healthcare facilities situated in rural communities in the KZN province of South Africa. All the selected facilities offer VMMC services and have an estimated population of 110 000–250 000 men in the age group of 15–49 years. Each facility performs around 700–2800 VMMCs on boys and men annually, with the majority of them being young boys between the age of 10 and 14 years.

### Sampling and recruitment

Purposive sampling was used to select the health facilities and participants for data collection. The health facilities were selected on the basis of sociocultural diversity and the performance of VMMC annually. The men who formed part of the study were recruited through mediated access (wherein staff within the facility were approached to have access to participants), while waiting to be attended to at the outpatient department in the selected facilities. Participants who voluntarily consented to participation and met the eligibility criteria (male candidates of 18 years and older and having undergone medical circumcision) for the study were selected to be part of the study. Data were collected from participants after they had been attended to at the health facility.

### Data collection

Data were collected between January and March 2019 following ethical approval from the Biomedical Research Ethics Committee of the University of KwaZulu-Natal (BE 627/18). Voluntary informed consent was obtained verbally and in writing from participants prior to data collection. Individual, in-depth interviews were conducted to gather data by using a semi-structured interview guide that contained a demographic section and questions designed to draw out circumcised men’s perceptions, understanding and experiences of VMMC. The interview schedules were written in English and translated into Isizulu. All interviews were conducted in Isizulu, transcribed and then translated verbatim into English by a language specialist. Private consulting rooms within each of the health facilities were used to conduct interviews privately, each lasting approximately 25–35 min. Data were collected until saturation was reached.

### Data analysis

After data collection, the audio-recorded interviews were transcribed verbatim and then analysed following phenomenographic data analysis procedures. The analysis of data was an iterative process following a step-wise method as recommended by Sjöström and Dahlgren.^24^ Firstly, the interview transcripts were read several times while listening to the audiotapes to ensure that data were transcribed accurately, and also to gain overall insight into the data. The second step entailed a more focused reading so as to obtain similarities and differences from the data. Thirdly, meaningful aspects of the transcript were extracted. The fourth step involved a preliminary grouping of similar responses leading to the development of an initial list of descriptive categories that were later refined by constant comparison with the transcript. The naming of the final set of categories was performed and the outcome space was formulated, based on the internal relations between the categories of description.

Data analysis was conducted in collaboration with an expert in qualitative research methods. The results of the study are presented in categories of description of participants’ perceptions, understanding and experiences of VMMC in KZN, South Africa.

### Ethical considerations

This study forms part of a larger study conducted to analyse the qualitative differences in primary healthcare stakeholders’ experiences, understanding and conceptions of VMMC in KwaZulu-Natal. Ethical approval to conduct this study was obtained from the Biomedical Research Ethics Committee of the University of KwaZulu-Natal (BE 627/18). Informed consent was obtained verbally and in writing from the participants prior to data collection. A written information sheet was provided to the participants clarifying the nature of the study, and written consent was sought from the participants after verbal approval was obtained.

## Results

### Sociodemographic data

A total of 12 participants formed part of the study; however, data saturation was reached by the 10th participant, and the additional two participants were added to ensure depth of the research findings. The participants were men aged 18–49. Most participants were above the age of 25 (*n* = 8), although the others were between the age of 18 and 25. The older participants had little to no formal education although the younger participants had at least completed high school and some had attained a tertiary-level education. Pseudo-names were used to present the data obtained from participants. The demographic details of the participants are shown in Table 1.

**TABLE 1:** Profile of participants.

Participant	Age	Highest level of education	Ethnic group	Religious and/or cultural belief	Duration circumcised	Relationship status
Thabo	29	College diploma	Black African	New testament Christian	5 years	In a relationship
Nathi	38	College diploma	Black African	Old testament Christian	7 years	Married
Mthobisi	24	Grade 12	Black African	New testament Christian	2 years	In a relationship
Lucky	21	Grade 12	Black African	New testament Christian	1 year	In a relationship
Xolani	32	College diploma	Black African	African traditional religion	4 years	In a relationship
Ayanda	23	College higher certificate	Black African	Old testament Christian	1 year	In a relationship
Makhehla	42	Grade 10	Black African	African traditional religion	6 months	Married
Zamokwakhe	39	College certificate	Black African	African traditional religion	8 years	Married
Siyanda	27	College higher certificate	Black African	Old testament Christian	2 years	In a relationship
Mthokozisi	19	Grade 12	Black African	Old testament Christian	1 year	In a relationship
Nduduzo	37	Grade 12	Black African	Old testament Christian	3 years	Married
Sandile	35	College certificate	Black African	New testament Christian	4 months	In a relationship

The findings are presented in categories of description that emerged from participants in relation to perceptions, understanding and experiences of VMMC in KZN, South Africa. The categories of description are listed in Table 2, which are as follows.

**TABLE 2:** Summary of categories of description emerging from participant interviews.

Perceptions	Understanding	Experiences
**Category 1**		
Personal health benefit	HIV prevention and medical circumcision	Delayed delivery of care
**Category 2**		
Perceived indirect partner benefits	Healing time vs. VMMC time	Pain, discomfort and poor wound healing
**Category 3**		
Modern procedure	Traditional vs. medical circumcision	Improved sexual performance vs. reduced penile sensitivity

HIV, human immunodeficiency virus; VMMC, voluntary medical male circumcision.

### Perceptions of voluntary medical male circumcision in KwaZulu-Natal

#### A personal health benefit

Participants viewed VMMC as a procedure that provided them with health benefits in terms of HIV prevention, reducing the chances of attaining sexually transmitted infections (STIs) and promoting general penile hygiene. These men also reported that such benefits potentially result in improving the quality of life in that the complications of ill health associated with HIV infection and other STIs are reduced. Seven male participants in this study revealed that this was the reason for undergoing VMMC.

‘I was made aware of the diseases, more especially [at] a time where I could be affected by diabetes. It would have a negative impact on my life if I were not circumcised. This is because a wound would start to develop on my private part.’ (Makhehla, 42 years, black African)

‘What changed my mind was that I heard about the 60% chance of not contracting HIV/AIDS.’ (Siyanda, 27 years, black African)

‘When you are circumcised, you reduce the chances of being infected with STIs or STDs [sexually transmitted diseases] as well as HIV/AIDS.’ (Thabo, 29 years, black African)

#### Perceived indirect partner benefits

Male candidates in this study also viewed medical circumcision as having indirect benefits for female partners in terms of health in that the procedure was seen to also potentially help women not to contract certain infections that might easily be transmitted when a man has a foreskin.

‘I think that medical circumcision is good because it also benefits the female partner [so that she does not] get infections easily which are normally found more in a penis with [a] foreskin.’ (Mthokozisi, 19 years, black African)

‘Women also get benefits from a man who has done medical circumcision because it helps the man to be healthier down there, meaning that she may also be healthier depending on how she carries herself.’ (Nathi, 38 years, black African)

‘I can say that medical circumcision helps a woman indirectly because a foreskin has a lot of dirt and when it’s removed, the man becomes cleaner and unable to infect her with any dirt so she is prevented from getting certain diseases.’ (Lucky, 21 years, black African)

#### Modern procedure

Participants were of the view that the medical approach to circumcision is a modern-day practice for a young boy or man who wants to keep abreast of the times. They also expressed the opinion that such a procedure is relevant for the older man who is promiscuous or desires to prove his sexual abilities.

‘I don’t think this procedure is very important when you are old unless you are at risk of developing diabetes, or you are a man who sleeps around a lot, then you need to help you in preventing HIV infection.’ (Makhehla, 42 years, black African)

‘My view is that young boys and men should do this thing because they are sexually active and there are a lot of diseases now in this age, so it will help then since young ones like sleeping around.’ (Zamokwakhe, 39 years, black African)

‘I have heard that once someone is circumcised the women say that person performs well in bed, so, since the young boys like doing this, it would work best for them and someone who wants to please their partner.’ (Mthobisi, 24 years, black African)

### Understanding of voluntary medical male circumcision in KwaZulu-Natal

#### Human immunodeficiency virus prevention and medical circumcision

Participants in this study expressed accurate knowledge about the partial protection offered by VMMC against HIV infection. A few did, however, misunderstand this, expressing the opinion that medical circumcision provided full protection against STIs, resulting in them not practising safe sexual intercourse.

‘Medical circumcision helps reduce the chance of getting HIV by 60%.’ (Xolani, 32 years, black African)

‘It protects one from getting certain STIs and HIV/AIDS.’ (Ayanda, 23 years, black African)

‘Medical circumcision provides protection from illnesses such as HIV and other STDs. Since I have [been] circumcised, I have proven it when I slept with a girl without using a condom and this girl is a loose one from the neighbourhood, after that I didn’t get a drop and all that; I was just fine.’ (Nduduzo, 37 years, black African)

#### Healing time versus voluntary medical male circumcision time

Participants in this study revealed that the best time to undergo medical circumcision would be when one is young; the understanding expressed is that healing occurs faster when one is younger. Participants also stated that the season during which one undergoes circumcision also has an influence on the healing process; men in this study believed that certain seasons of the year promote quicker healing. In this study, about half of the participants revealed that summer would be the best time to be circumcised because they felt that one heals quicker in summer. The other six believed that winter is the best time to do VMMC because there is a perceived lower chance of the wound becoming infected; participants also expressed a belief that the weather conditions in winter make it unfavourable for bacteria to infect the wounds.

‘It is cold in winter; I think perhaps the healing process is different compared to summer which I think will promote healing.’ (Makhehla, 42 years, black African)

‘The best time for one to do circumcision is as young as possible, even like [at] 8 or 10 years old. I think that the younger you are the quicker you heal.’ (Sandile, 35 years, black African)

‘I think that one should do circumcision when it’s winter because, in summer, you may experience infection of the wound because of the higher temperatures; also, the younger you are, the better, because your body is functioning better and will heal better.’ (Lucky, 21 years, black African)

#### Traditional versus medical circumcision

In this study, participants understood the difference between medical and traditional circumcision in terms of the nature of both procedures. The majority of participants understood the cultural significance of traditional circumcision, although they also communicated an accurate understanding of the role of medical circumcision in the context of HIV infection and promoting general positive health outcomes. Certain participants expressed that although medical circumcision is appropriate as a health intervention, it does not replace the cultural value of traditional circumcision.

‘I think traditional and medical circumcision are completely different because the former is done by someone who is just an elder and [who] does not have any training [concerning] the human body, whereas the medical one is done by someone who is educated about health matters and is therefore safer.’ (Makhehla, 42 years, black African)

‘The medical one involves removing the foreskin in a more controlled, safe way because the procedure is conducted in a clean environment with adequate back-up should things go wrong.’ (Thabo, 29 years, black African)

‘I think that medical circumcision is important today because of all the diseases it helps to prevent. The only thing is that they don’t teach all the traditional values that are taught when someone does it in the mountains.’ (Nathi, 38 years, black African)

### Experiences of voluntary medical male circumcision in KwaZulu-Natal

#### Delayed delivery of care

Participants reported that the waiting time to undergo a medical circumcision had been long, at least about 2 h, excluding the preoperative care that they had performed before the procedure (HIV testing, comprehensive health screening, VMMC counselling and consent). These participants stated that they either waited because the service providers who were scheduled to come had not arrived in time, or because the numbers of men and boys who had reported to undergo medical circumcision had been high.

‘I came at around 8 a.m., opened a file and did all the check-ups and was told to wait for the people who were coming to do the circumcision. They took a while because these people only got there around 12 o’clock, if I am not mistaken.’ (Mthokozisi, 19 years, black African)

‘… They got here very late; I think before they came we had already even [been] given food.’ (Ayanda, 23 years, black African)

‘There were a lot of delays mainly because there were many of us, there were also school children that had come with a mini-bus.’ (Sandile, 35 years, black African)

#### Pain, discomfort and poor wound healing

Certain participants reported experiencing mild to moderate pain and discomfort during the procedure. Participants in this instance believed that such discomfort could have been because of the wearing-off of the anaesthesia, as the waiting period between receiving the injection and being circumcised had been unusually long. This increase in the waiting period was attributed to the shortage of people performing what the participants termed the actual cutting. The men in this study also experienced pain and discomfort during the postoperative healing period, related to the nature of the wound and, in certain cases, stemming from a failure to adhere to wound care procedures that they had been taught at the clinic.

‘I don’t want to lie, the injection started to wear off a little. Another thing, maybe if I had gone straight to the doctor immediately after the injection, maybe I would also be one of those that did not feel pain.’ (Sandile, 35 years, black African)

‘The lower part of my penis took longer to heal and resulted in me experiencing certain pain. Even after six weeks it was still painful.’ (Lucky, 21 years, black African)

‘I had problems with the stiches and pain especially when I would get an erection. It would be as if the stitches would burst … [the] night time was the worst and early in the morning the discomfort would be there.’ (Siyanda, 27 years, black African)

#### Improved sexual performance versus reduced penile sensation

Male candidates reported a perceived improvement in their sexual performance, particularly in terms of the duration of sexual intercourse. These participants also reported that their partners had informed them regarding their improved performance and increased sexual pleasure. Although participants perceived their sexual functioning to have increased, most reported that the degree of pleasure that they had been receiving was reduced because of a perceived decrease in penile sensitivity that they experienced. These participants were, however, more pleased by the fact that they had improved for their female partners.

‘When I had the foreskin I would ejaculate early, but now I take time to ejaculate, so I can say that I got an upgrade in this way, because on social networks girls say they don’t like a person who ejaculates early.’ (Thabo, 29 years, black African)

‘Right now, I can perform until I get tired and I find that I won’t ejaculate easily, so, in other words, I’m more powerful and my lady likes that. As a man you [also] want to feel that you are powerful and pleasing your person.’ (Zamokwakhe, 39 years, black African)

‘The difference is that when I still had this thing (the foreskin), there was that feeling and now it’s like it’s lost when the foreskin was removed.’ (Ayanda, 23 years, black African)

#### Decision to undergo voluntary medical male circumcision

The findings of this study revealed that the decision to undergo VMMC was one that was complex and resulted from a combination of individual intrinsic and extrinsic factors. The intrinsic factors consisted mainly of an inner desire and general admiration for the procedure and individuals who have performed it. The extrinsic factors are the information received from influencers who, in this study, are close friends, family members and peers. In this study, peer groups, social media and female partners were found to play a huge role, both in terms of providing information that facilitated the motivation to undergo VMMC, and also the general psychosocial support system. From the information gathered from participants, the decision to undergo VMMC was one which occurred in the region of 8 months to 3 years from the time a man had first received information about the procedure to when he actually underwent VMMC.

‘I first heard about medical circumcision in 2009 from my close friends who were guys … they had heard from the radio that medical circumcision had many benefits to men and women, some of my family members also encouraged me to do because of the health benefits so I decided to do it 2012.’ (Thabo, 29 years, black African)

‘When I heard I about the benefits of medical circumcision in 2013 and saw my friends doing it, I liked it and thought I should also do it, the problem was getting the time off from work to do it but I liked it so I eventually made a plan in 2017.’ (Siyanda, 27 years, black African)

## Discussion

The aim of this study was to analyse circumcised men’s perceptions, understanding and experiences of VMMC in KZN, South Africa. The study findings revealed that circumcised men’s perceptions of VMMC were centred on the direct and indirect health benefits of the procedure. Participants’ understanding of VMMC was in relation to the HIV prevention and the nature of traditional and medical circumcision. The experiences of participants were on the quality of care received during the procedure and the effect of medical circumcision during and after VMMC surgery.

Most participants in this study revealed that they conceived VMMC to have personal health benefits, particularly in terms of HIV prevention and reduction of STIs. In this study, this was found to be one of the major motivators for men to undergo medical circumcision. Other studies concur with this finding in terms of circumcised men’s beliefs about protection against HIV infection.^26,27,28^ However, a false sense of complete protection against HIV infection is of concern, as it may result in unsafe sexual practices.

Other perceptions of VMMC in this study were related to the perceived indirect partner benefits of VMMC. Participants in this instance revealed that they perceived VMMC to also potentially result in better health outcomes for their female partners through indirect prevention of certain STIs by virtue of improved penile hygiene following male circumcision. This was also another motivator for men to undergo VMMC. Similarly, in other settings, it has been found that women play an important role in influencing uncircumcised men to seek medical circumcision.^29,30^ The findings of this study, which are supported by previous findings, highlight the need for the role of women to be explored in relation to the uptake of VMMC by male candidates, so that maximum coverage of medical circumcision can be attained.

Certain participants in this study conceptualised VMMC as a modern procedure for mainly young men who want to keep abreast of the times. This finding is indicative of the social tensions that exist between the medical and traditional practice of male circumcision. A previous study on the acceptability of medical circumcision within a South African community that practises traditional circumcision revealed that men are largely opposed to the medical approach to male circumcision.^15^ Another study has indicated that circumcision is an important rite of passage in most cultures, and therefore scaling VMMC in the context of tensions between traditional and medical practice requires a balance between tradition and the health benefits of medical circumcision.^31^ In such instances, support for VMMC from traditional leaders is instrumental.

In terms of men’s understanding pertaining to VMMC, most participants expressed accurate knowledge about the nature of partial protection. A few did, however, appear to have a degree of misunderstanding about the protection offered by VMMC against HIV infection and other STIs. Previous studies have shown that medical male circumcision could prevent female-to-male transmission of HIV infection by up to 60%. Other studies have revealed lower incidences of STIs, penile cancers and carcinomas among medically circumcised men.^32^ The accurate knowledge of the majority of the participants regarding the partial protection of VMMC against HIV infection and other STIs is potentially beneficial, as it suggests that these male candidates may be receptive to a combination of other HIV preventive strategies such as condom use, thereby further decreasing their chances of acquiring HIV infection. However, the poor understanding of certain participants regarding partial protection may imply that these men are more likely to engage in risky sexual behaviours.

Regarding healing time and VMMC time, participants were divided in terms of their response. In general, participants seemed to be of the understanding that specific seasons of the year are the times to perform medical circumcision as these are perceived to promote healing based on several assumptions. Pertaining to healing time, most participants reported being of the understanding that healing post-circumcision takes place quicker when one is younger. This finding indicates a misunderstanding and misperception of the nature of VMMC, which has a potential impact on the acceptability and uptake of VMMC.

Most men in this study understood the difference between traditional and medical circumcision in terms of the nature of both procedures and the cultural significance of traditional circumcision. This finding is important as it provides a foundation upon which information regarding the nature of VMMC can be given. The findings of a qualitative study to address the barriers to the uptake of VMMC in Kenya have revealed that men require accurate and detailed information concerning what to expect during VMMC.^33^

Delayed delivery of care was an experience reported by most participants who had undergone VMMC at a primary healthcare facility in KZN. The circumcised men reported that delays were because of the high demand for VMMC service which was worsened by the shortage of staff and the late arrival of staff during VMMC campaigns. A recent study on the behaviour change pathways to VMMC has also shown how the implementation of VMMC is challenged by service delivery shortfalls, meaning that the full health impact of clients who are ready to undergo VMMC is almost always compromised.^34^ An earlier study on matching the supply and demand for VMMC in a high-volume campaign in Tanzania has shown that through the adoption of task shifting and task sharing and various approaches to conducting medical circumcision, the demand for VMMC could be met by a supply of services.^35^

The experiences of minor pain and discomfort during the procedure were also reported by a significant number of participants. Participants had different explanations for the pain they experienced. The apprehension of pain and discomfort during VMMC surgery has been cited as one of the major barriers to the uptake.^22,36,37^ In addition to providing education to address this issue, the issue of pain intra-operatively should be dealt with through the effective provision of analgesia during and after the procedure, and the education of clients on the self-management of the wound to prevent and manage pain that may arise during the healing period.

A significant number of participants also reported a perceived enhanced sexual performance following medical circumcision; however, these participants also stated that they experienced a reduction in penile sensitivity after the procedure. Participants were, however, more pleased with the fact that their sexual performance had improved after undergoing VMMC. A study on the effect of circumcision on the sexual functioning of adults revealed that ejaculatory latency time increased following medical circumcision, and was found to be an advantage in this study.^38^ In recent acceptability studies on VMMC, improved sexual performance has been cited as a motivator for VMMC.^22,23^

In this study, it was found that the decision to undergo VMMC was a complex psychosocial process influenced by many individual intrinsic and extrinsic factors. This finding is significant as it provides the basis upon which demand generation interventions can be based and also highlights the complexity of the VMMC decision-making process that has to be understood when tailoring messaging for demand generation.

### Limitations

The study was limited because of the sample size and the choice of research design, which does not allow for the findings to be generalised to a larger population. In addition, the choice of individual interviews as the only means of data collection could have compromised the depth and quality of results attained. However, the findings do provide important baseline data on the motivators and possible barriers to the uptake of VMMC in an adult population, in the context of a low-income rural setting that is influenced by culture and tradition.

## Conclusion and recommendations

The study findings suggest that various contextual factors influence male client’s perceptions and understanding regarding VMMC. The decision to undergo VMMC is one which is complex, often taking long time periods, influenced by an array of factors such as religion, tradition, societal norms, female partners and the structure of the healthcare system. In this study, it was found that both correct and incorrect perceptions and understanding regarding VMMC served as the motivators for male candidates to undergo VMMC. The correct perceptions provide an entry point upon which VMMC demand creation interventions should be based; however, the misperceptions and misunderstanding serve as a potential threat to the overall long-term health benefits of VMMC. Participants’ experiences of VMMC seem to suggest that the healthcare system is yet to fully comprehend the health needs of men in the light of their contextual influences. The negative experiences reported in this study are potential barriers to uptake, which need to be addressed.

These findings have important implications for health policy and practice as they suggest a need for messaging and practice interventions to take cognizance of the contextual factors that influence perceptions and understanding around VMMC. Fostering multi-sectoral collaborations at community level and contextually sensitive interventions at an individual and family level could be the first step to attaining acceptability of VMMC. Participants’ experiences, in terms of the quality of care rendered, could be addressed through specific changes in the policy-guiding practice of VMMC; such changes could include adoption of proven cost-effective and safe techniques of conducting medical circumcision. In addition, Healthcare workers should be given regular in-service training on the provision of safe, effective and efficient VMMC. The provision of comprehensive HIV prevention services including, but not limited to, pre- and post-VMMC counselling should also address behavioural aspects post-VMMC to prevent risky sexual practices.
